# Evaluation of the external validity of a joint structure–function model for monitoring glaucoma progression

**DOI:** 10.1038/s41598-020-76834-4

**Published:** 2020-11-12

**Authors:** Sampson Listowell Abu, Mahmoud Tawfik KhalafAllah, Lyne Racette

**Affiliations:** 1 Department of Ophthalmology and Visual Sciences, University of Alabama at Birmingham, Birmingham, AL 35233 USA; 2 Vision Science Graduate Program, University of Alabama at Birmingham, Birmingham, AL 35233 USA

**Keywords:** Eye diseases, Optic nerve diseases, Diseases

## Abstract

The dynamic structure–function (DSF) model was previously shown to have better prediction accuracy than ordinary least square linear regression (OLSLR) for short series of visits. The current study assessed the external validity of the DSF model by testing its performance in an independent dataset (Ocular Hypertension Treatment Study–Confocal Scanning Laser Ophthalmoscopy [OHTS–CSLO] ancillary study; N = 178 eyes), and also on different test parameters in a sample selected from the Diagnostic Innovations in Glaucoma Study or the African Descent and Glaucoma Evaluation Study (DIGS/ADAGES). Each model was used to predict structure–function paired data at visits 4–7. The resulting prediction errors for both models were compared using the Wilcoxon signed-rank test. In the independent dataset, the DSF model predicted rim area and mean sensitivity paired measurements more accurately than OLSLR by 1.8–5.5% (p ≤ 0.004) from visits 4–6. Using the DIGS/ADAGES dataset, the DSF model predicted retinal nerve fiber layer thickness and mean deviation paired measurements more accurately than OLSLR by 1.2–2.5% (p ≤ 0. 007). These results demonstrate the external validity of the DSF model and provide a strong basis to develop it into a useful clinical tool.

## Introduction

Early detection of glaucoma progression is crucial to preserve vision^1,2^ yet it remains a challenging multi-factorial issue^3^. Without diligent assessment and proper interpretation of clinical data, the presence of glaucoma progression can be elusive. The tests and strategies for monitoring progression are becoming increasingly objective and reliable^4,5^. Additionally, several mathematical models have been developed to aid with the interpretation of clinical data and decision making. These models include fundamental regression analysis^6,7^ and complex statistical computations such as Bayesian approaches^8–10^ and machine learning strategies^11–13^. Although very promising, most of these novel models have yet to be integrated into routine clinical practice.

To be clinically relevant, models must be generalizable to different populations, conditions, tests and parameters^14–16^. This makes external validation, the process of assessing a model’s performance in an independent dataset^17–20^, a crucial step in the development of models in clinical research. The problem, however, is that the majority of these models are not externally validated^17,19^ and a handful may have undergone this step but with some limitations. For instance, among the few validated models in glaucoma research^20–24^, two were validated with a sample size smaller than that recommended for external validation^21,22^ and three resampled the original dataset instead of using an independent dataset^20,21,23^. The use of a smaller sample and/or a subset of the original dataset may result in an overestimation of the model’s performance^19,20^. Collin et al. recommended a minimum sample size of 100 for external validations, and preferably 200 events^17^. Similarly, Vergouwe and colleagues suggested a minimum of 100 events and 100 non-events as a reasonable sample size for external validation^25^.

We previously developed the dynamic structure–function (DSF) model to identify glaucoma progression^26^. Instead of combining structural and functional information into a univariate metric of progression^8,10^, the DSF model assesses structural and functional change jointly in a two-dimensional space. The predictive performance of the DSF model has been assessed in 220 eyes with either ocular hypertension or primary open-angle glaucoma (POAG) selected from the Diagnostic Innovations in Glaucoma Study (DIGS) or the African Descent and Glaucoma Evaluation Study (ADAGES)^27^. In comparison with the ordinary least square linear regression (OLSLR) model, the DSF model made significantly more accurate prediction of rim area (RA) and mean sensitivity (MS) paired measurements for short series of up to 7 visits^26^.

This encouraging finding prompted the need to determine whether the DSF model could yield similar results when tested in different populations, and with different tests and parameters. The present study was designed to evaluate the external validity of the DSF model. Using an independent dataset from the Ocular Hypertension Treatment Study–Confocal Scanning Laser Ophthalmoscopy (OHTS–CSLO) ancillary study^28^, the prediction error (PE) resulting from the prediction of global and sectoral RA–MS paired measurements was compared between the DSF model and OLSLR. We paired RA and MS in order to be consistent with the structure–function pairs used in the study that we seek to validate^26^. To determine the applicability of the DSF model to different parameters, we compared PE obtained for the joint prediction of retinal nerve fiber layer thickness (RNFLT) and mean deviation (MD) from a resampled cohort of POAG eyes enrolled in the DIGS and ADAGES studies. RNFLT and MD were considered for this analysis because they are the most common and sensitive structural^29,30^ and functional^31,32^ parameters used by clinicians to monitor glaucoma progression. The objective here was to ascertain whether the DSF model will perform well with new tests and parameters that will emerge as clinically useful in the future.

## Results

### Validation of the DSF model in an independent (OHTS–CSLO) dataset: prediction of RA and MS paired measurements

**Figure 1:**
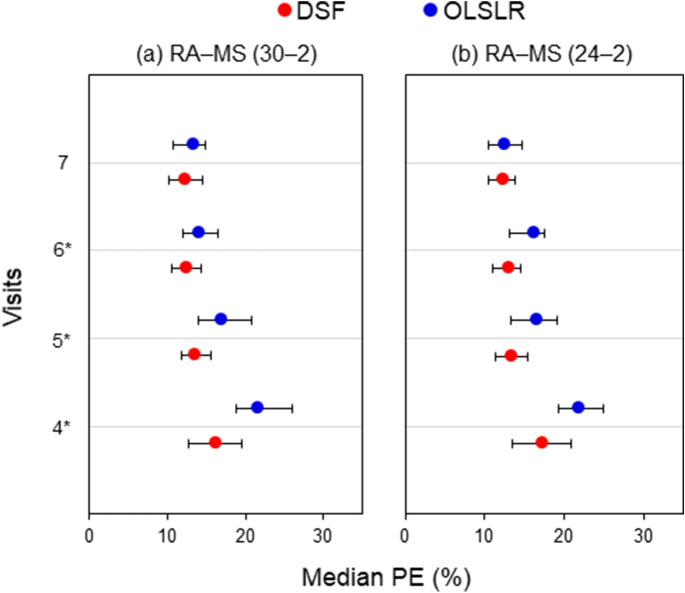
Median PE and 95% confidence intervals obtained for the DSF model (red circles) and the OLSLR model (blue circles) for the prediction of RA–MS paired measurements from the 4th to 7th visit. Panels *a* and *b* represent predictions made with MS computed from the 30–2 SAP test pattern and from the 24–2 SAP test pattern, respectively. Asterisks denote visits where the DSF model had significantly lower PE than OLSLR.

Figure 1 shows the median PE obtained for the DSF and OLSLR models for the prediction of global RA and MS paired measurements. When RA was predicted jointly with MS derived from the 30–2 static automated perimetry (SAP) test pattern, the median PE obtained for the DSF model was significantly lower (1.8–5.5%, p ≤ 0.004) than for OLSLR at the 4th–6th visits (Fig. 1a). For the joint prediction of RA with MS computed from the 24–2 SAP test pattern (Fig. 1b)*,* the median PE for the DSF model was significantly lower (3.2–4.8%, p ≤ 0.001) than for OLSLR at the 4th–6th visits. For both types of RA–MS pair, the difference in median PE between the two models was not significant at the 7th visit. On average, the DSF model had lower PE than the OLSLR model in 72% of the eyes at visit 4, in 67% at visit 5, in 62% at visit 6 and in 53% at visit 7.

**Figure 2:**
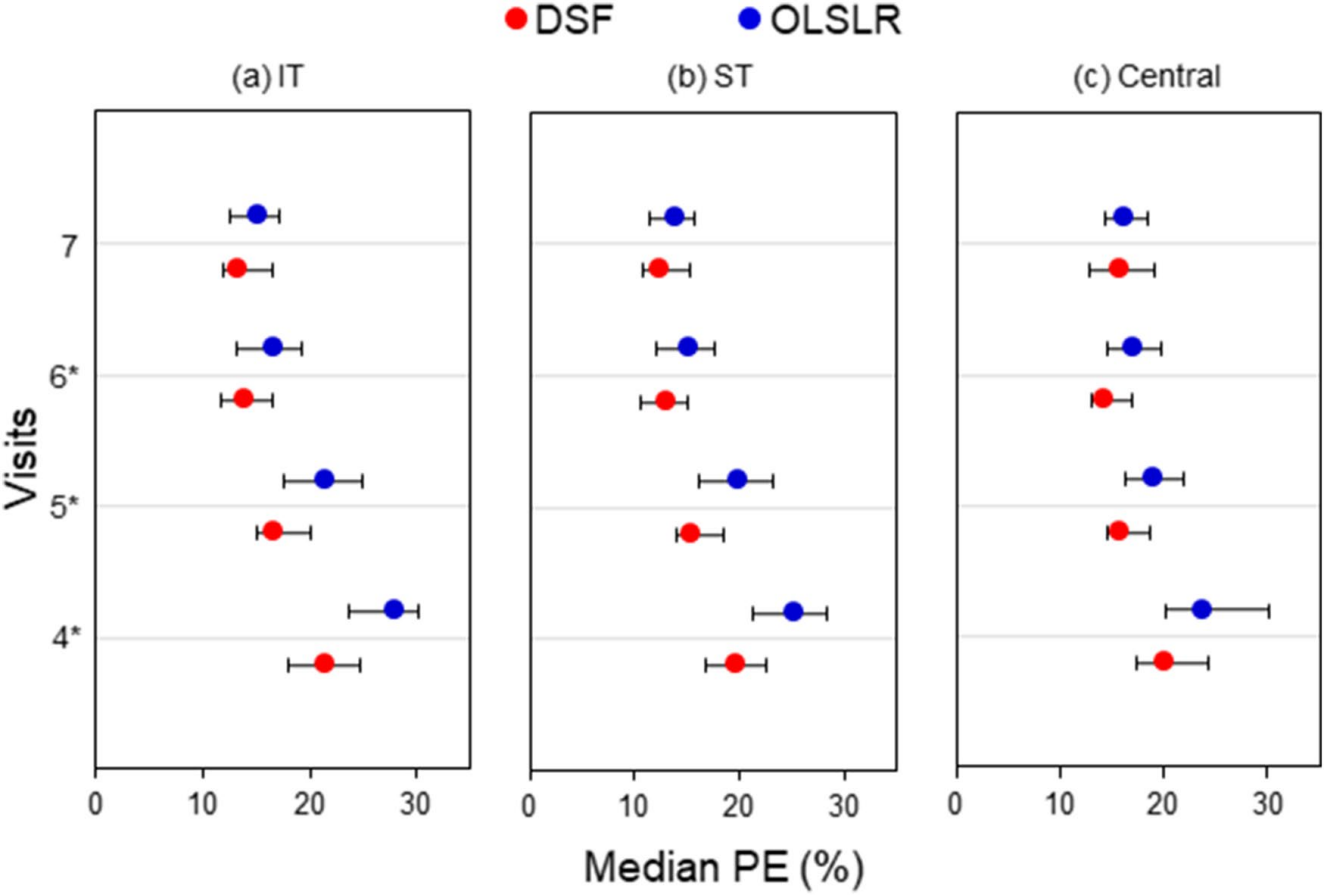
Median PE and 95% confidence intervals obtained for the DSF model (red circles) and the OLSLR model (blue circles) for the prediction of sectoral RA–MS paired measurements from the 4th to 7th visit. Panels *a, b* and *c* represent predictions for the infero-temporal (IT) sector, supero-temporal (ST) sector, and Central region, respectively. Asterisks denote visits where the DSF model had significantly lower PE than OLSLR.

Figure 2 shows comparisons of median PE between the two models for the prediction of sectoral RA and MS paired measurements. Except for predictions at the 7th visit, the median PE obtained for the DSF model was significantly lower (2.1–6.4%, p ≤ 0.002) than for OLSLR in all sectors considered.

### Validation of the DSF model with different parameters in the DIGS/ADAGES: prediction of RNFLT and MD paired measurements

**Figure 3:**
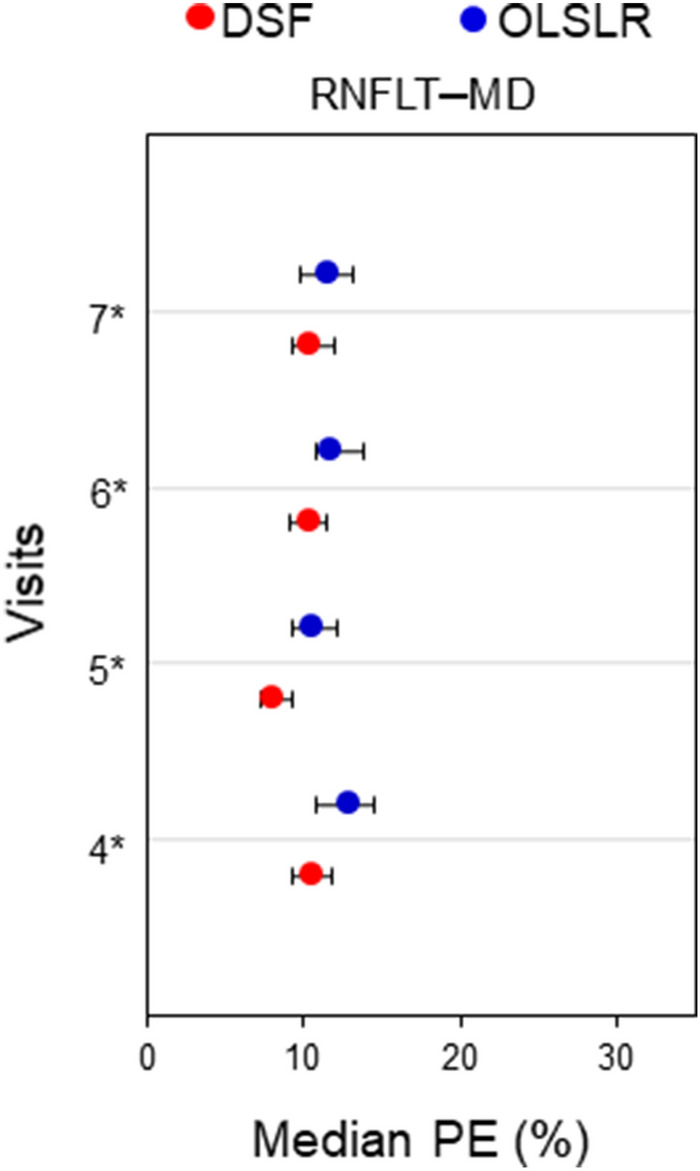
Median PE and 95% confidence interval obtained for the DSF model (red circles) and the OLSLR model (blue circles) for the prediction of RNFLT–MD paired measurements from the 4th to 7th visit. Asterisks denote visits where the DSF model had significantly lower PE than OLSLR.

Figure 3 shows comparisons of median PE between the DSF and OLSLR models for the prediction of RNFLT and MD paired measurements. From the 4th to 7th visit, the median PE obtained for the DSF model was significantly lower than for OLSLR by 1.2–2.5% (p ≤ 0. 007). The DIGS/ADAGES dataset included 393 POAG eyes which were previously subclassified at baseline into glaucomatous optic neuropathy only (GON-alone; 121 eyes), glaucomatous visual field only (GVF-alone; 97 eyes) and those with both GON and GVF (175 eyes)^27^. Table 1 presents the comparison of median PE between both models for the three baseline classifications of POAG eyes. In eyes with GON only, the DSF model had significantly lower PE than OLSLR across all visits. Similar results were obtained in eyes with both GON and GVF, except at the 7th visit where there was no significant difference in PE between both models. In eyes with GVF only, while the PE was always lower than that of OLSLR, statistical significance was reached only at visit 5. The DSF model made more accurate prediction in a greater proportion of eyes than OLSLR (19 – 39% more for GON-alone eyes, 13 – 32% more for GVF-alone and 14 – 36% more for eyes with both GON and GVF).

**Table 1:** Comparison of median PE between the DSF model and OLSLR for three baseline classifications of POAG eyes.

	GON-alone eyes N = 121			GVF-alone eyes N = 97			Both GON and GVF N = 175		
	DSF	OLSLR	p value	DSF	OLSLR	p value	DSF	OLSLR	p value
4th visit	11.19	13.37	< 0.01	10.67	12.99	0.070	10.67	13.02	< 0.01
5th visit	8.44	11.17	< 0.01	8.22	10.96	0.001	8.29	11.14	< 0.01
6th visit	10.59	12.29	0.002	10.62	11.98	0.269	10.53	12.23	0.005
7th visit	11.18	11.89	0.015	10.46	11.65	0.644	11.08	11.65	0.079

## Discussion

Several mathematical models have been developed with the goal of improving the understanding of disease trajectories and also to aid clinical decision making^19^. The majority of such models, however, do not make inroads into clinical practice partly due to their lack of external validity^18,19^. In this study, we assessed the generalizability of the DSF model which has been reported to have better predictive accuracy than the OLSLR model for series of 4–7 visits^26^. We assessed the performance of the DSF model in an independent dataset^28^, and on different test indices (RNFLT and MD), and found that it predicted subsequent structure–function paired measurements more accurately than OLSLR for short series of up to 7 visits.

Our results show that the DSF model can be generalized to different populations and test indices. This provides the basis to further develop the DSF model into a useful clinical tool for detecting and/or predicting glaucoma progression. The DSF model’s superior predictive ability over short follow-up series also alludes to the possibility of applying it to assess progression when limited data is available. This may lead to earlier detection of progression and inform clinical decisions to stop or slow vision loss. However, at present, the DSF model lacks the ability to make a determination of the progression of status of an eye. Developing it into a clinical tool, will involve two crucial steps. The first step is to incorporate a robust statistical test into the DSF model to evaluate change in predicted measurements. The next step is to establish how the model’s sensitivity compares to that of conventional methods used for assessing progression. The present study expanded the analysis beyond predicting global structure–function paired measurements by comparing the performance of both models in estimating sectoral measurements. The DSF model predicted sectoral RA and MS paired measurements more accurately than OLSLR (Fig. 2).

Recent advances in ocular imaging, such as optical coherence tomography (OCT), have enhanced our ability to assess the optic nerve head and different retinal layers. The OCT-derived RNFLT has better sensitivity to detect early glaucomatous changes than the Heidelberg Retinal Tomograph (HRT)-derived RA^33–35^; hence it has been widely adopted in both clinical and research settings. To ascertain whether the DSF maintains its performance for different structure–function parameters, RNFLT was predicted jointly with MD for 393 POAG eyes selected from the DIGS/ADAGES dataset. We found that the median PE obtained for the DSF model was significantly lower than that for OLSLR (Fig. 3). This finding is consistent with the results obtained with RA and MS, either in the present study or the previous one^26^. Furthermore, this finding suggests that the DSF model can be applied to other structure–function parameters that will eventually emerge as promising to identify change in glaucoma. Additional analyses also showed that the DSF model obtained lower median PE than the OLSLR for each POAG subclassification (Table 1) and had better prediction accuracy in a larger percentage of eyes. This finding supports the DSF model’s potential as a valuable clinical tool.

**Figure 4:**
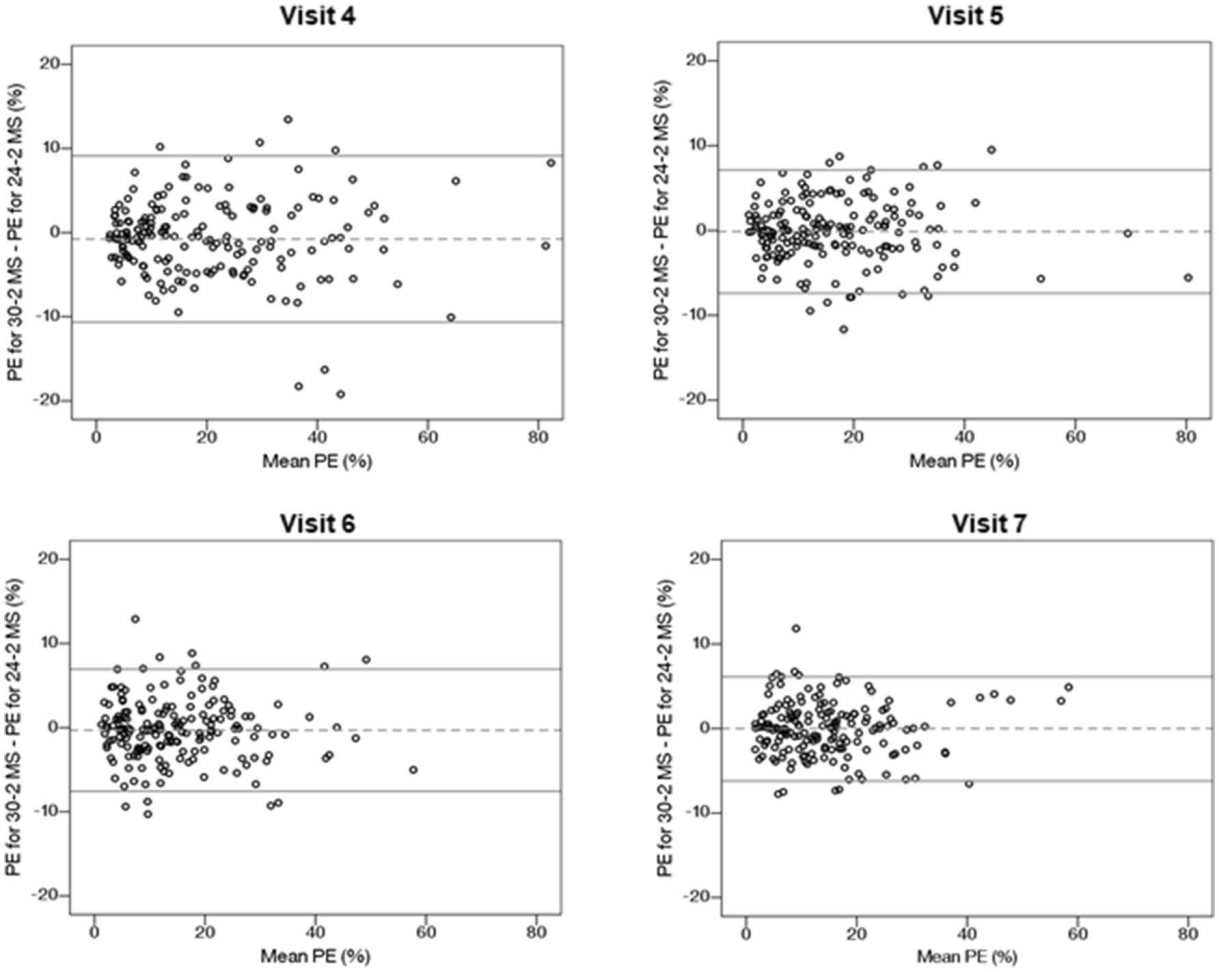
Bland–Altman plots showing the level of agreement between PEs resulting from the joint prediction of RA with 30–2 MS and with 24–2 MS using the DSF model. The horizontal axes represent mean PE and the vertical axes represent the difference in PE. The mean difference lines and corresponding 95% limits of agreement are shown as the dashed and solid lines, respectively.

Given that the detection of glaucoma progression is partly limited by measurement variability^3^, it is crucial to assess its impact on the performance of the DSF model. This impact was determined by comparing the median PE resulting from the joint predictions of RA with 30–2 MS (Fig. 1a) and with 24–2 MS (Fig. 1b). Heijl and colleagues found that, within the central 30° of the visual field, the threshold sensitivities in the periphery were significantly more variable than those in the midperiphery^36^. This suggests that the 30–2 test pattern may have more variable test indices (e.g. MS and MD) because it includes 22 additional test locations outside the area of the 24–2 test pattern. We found no statistically significant difference in the prediction accuracy of the DSF model when RA was predicted jointly with MS from either 30–2 and 24–2 test pattern (median PE difference = 0.08–1.1%, all *p* > 0.06). This observation is further illustrated with mean difference plots in Fig. 4. The closeness of the mean difference lines to zero suggests that the prediction accuracy of the DSF model was not adversely impacted by differences in measurement variability between the two tests. Ramezani et al*.* reported that the use of MS from contrast sensitivity perimetry, a test with lower test–retest variability than SAP, did not improve the prediction accuracy of the DSF model^37^. These observations suggest that measurement variability may have little or no impact on the performance of the DSF model.

This study has limitations. The first limitation is the potential misestimation of parameters in percent of mean normal given interindividual variations in structural measurements in healthy population^27^ coupled with the presence of floor effect^38,39^. Rescaling of parameters in percent of mean normal was, however, necessary in this study. The DSF, being a two-dimensional model, was applied to structural and functional components initially measured in different scales. In order to assess these different parameters jointly, we expressed them in a comparable scale. Another limitation is that RA measurements were rescaled based on normative data obtained from a different cohort of 91 healthy eyes described elsewhere^40^. This was necessitated by the unavailability of RA measurements taken at baseline in the OHTS study. Measurement of RA with HRT was later included in the OHTS protocol as an ancillary study^28^. The mean normal RA (1.44 mm^2^), computed from this separate dataset^40^, was within the range of average RA values (1.37–1.76 mm^2^) reported for healthy cohorts^41–44^. Of note, rescaling of the parameters was systematically applied to all participants and used to assess prediction accuracy in both models; hence any potential impact of the data source used to achieve this rescaling would have affected both models equally. Therefore, the quantification of parameters in percent of mean normal and the use of different normative datasets did not selectively influence the performance of one model over the other.

In conclusion, we assessed the external validity of the DSF model by determining its performance in an independent dataset and also with different parameters. Consistent with the previous study^26^, the DSF model had better prediction accuracy than OLSLR over short series of visits. The current study also showed that the performance of the DSF model is generalizable to different structure–function parameters. These results suggest that the DSF model has good external validity, is generalizable and has the potential to eventually be used as a clinical tool for early detection of glaucoma progression.

## Methods

### Study design

The present study was a retrospective analysis of two datasets to evaluate the external validity of the DSF model. An independent dataset, selected from the OHTS–CSLO ancillary study^28^, was used to assess the performance of the DSF model. The OHTS–CSLO data were released through a data access agreement signed on 01/23/2014. A resampled DIGS/ADAGES dataset was used to evaluate the performance of the DSF model with different parameters.

### OHTS–CSLO dataset

We selected 178 eyes of 105 patients (mean age: 53 ± 7 years) from the OHTS–CSLO ancillary (mean follow-up was 6.5 ± 0.6 years). The OHTS–CSLO study prospectively followed a cohort of ocular hypertensive patients with HRT (Heidelberg Engineering, GmbH, Dossenheim, Germany), disc photography and SAP 30–2 full-threshold test (Humphrey Field Analyzer, Zeiss, Dublin, CA, USA)^28^. The baseline paper details the eligibility and exclusion criteria used in the OHTS^45^.

#### RA and MS data for the current study

For the current study, we required each patient to have a minimum of 7 visits with at least 3 months between consecutive visits. We also required each patient to have reliable HRT and SAP tests at each visit. Unreliable HRT images, defined as a mean pixel height standard deviation > 50 μm, as well as unreliable visual fields, defined as false positives, false negatives, or fixation losses greater than 33%, were excluded. We used RA as the structural parameter and MS as the functional parameter. Each SAP test results in the OHTS–CSLO dataset had 76 sensitivity values in decibels (dB) which were converted into a linear unit (1/Lambert). After removing the test points above and below the blind spot, the remaining 74 test sensitivity values were averaged to obtain the 30–2 MS as described in Garway-Heath et al.^46^. To derive MS values for the central region, infero-temporal (IT) and supero-temporal (ST) sectors, we used the Garway-Heath map^46^. This map relates the 24–2 SAP pattern of test locations to the six HRT defined sectors of the optic disc. The central 52 test points of the 30–2 test pattern, which are consistent with the 24–2 SAP test pattern were extracted and then averaged accordingly to obtain 24–2 MS values for global, central region, IT and ST sectors. The MS for the central region, which corresponds to the temporal half RA of the optic disc, was computed as the mean of the sensitivity values of the central 16 test points^46^.

### DIGS/ADAGES dataset

We included 393 eyes of 254 POAG patients (mean age: 64 ± 10 years) selected from the DIGS or ADAGES cohort. Described in detail elsewhere^27^, the DIGS and ADAGES are multicenter longitudinal studies that enrolled and prospectively monitored retinal structure and function among healthy, glaucoma suspects and glaucoma patients. Eligibility criteria included one good quality stereoscopic photograph and a 24–2 SAP test at baseline, open angles, best-corrected acuity of 20/40 or better, spherical refraction within 5.0 diopters, and cylinder correction within 3.0 diopters, no history of intraocular surgery (except for uncomplicated cataract or glaucoma surgery), absence of comorbidities and use of medications that affect the visual field.

#### RNFLT and MD data for the current study

For the current study, we selected only patients with a POAG diagnosis at the DIGS/ADAGES baseline. Out of the 393 POAG eligible eyes, 121 had GON-alone, 97 had GVF-alone, and the remaining 175 had both GON and GVF^27^. In addition, we required each patient had a minimum of 7 visits, with RNFLT measurement taken with the Spectralis OCT (software version 5.2.0.3, Heidelberg Engineering, Heidelberg, Germany) and MD obtained with the 24–2 SITA Standard SAP test (HFA II, Carl Zeiss Meditec Inc., Dublin, CA). Visits had to be separated by a minimum of 3 months. The Imaging Data Analysis and Evaluation Reading Center, and the Visual Field Assessment Center at the Department of Ophthalmology, University of California, San Diego reviewed the quality and reliability of the OCT images and all visual fields^47^, respectively. Only OCT scans with signal strength greater than 15 dB and visual fields with less than 33% fixation losses, false negative and false positive were considered usable in the present study. The MD values were converted to linear units using the equation that Hood et al. previously applied to convert total deviation values to linear units (linear unit = 10^**MD/10**^)^38^.

### Rescaling structural and functional data to percent of mean normal

All measurements were rescaled to percent of the mean normal values^48,49^ to ensure that structural and functional data were quantified in a comparable scale. For the OHTS–CSLO dataset, the mean normal MS value was obtained from the normal OHTS baseline SAP tests. For the mean normal RA value, we used a separate dataset of 91 healthy eyes^40^. For the DIGS/ADAGES dataset, the mean normal values for both RNFLT and MD were derived from 395 healthy eyes selected from the DIGS/ADAGES using the selection criteria explained above. The mean normal value for each parameter is presented in Table 2. For healthy individuals with normal optic disc and intact vision, approximately 100% of mean normal is expected for RA and MS values. To exemplify the conversion to percent of mean normal values, we provide this example for a patient with POAG; with an RA of 1.05 mm2 and MS of 28 dB (630.96 1/L), the converted values will be 72.9% and 64.9%, respectively.

**Table 2:** Mean normal values used for rescaling of parameters.

Parameters	Mean normal value
RA (mm^2^)	1.44
RNFLT (µm)	98.47
MD (1/L)	1.06 (0.27 dB)
MS (1/L)	972.60 (29.90 dB)

### Prediction of structure–function pairs

**Figure 5:**
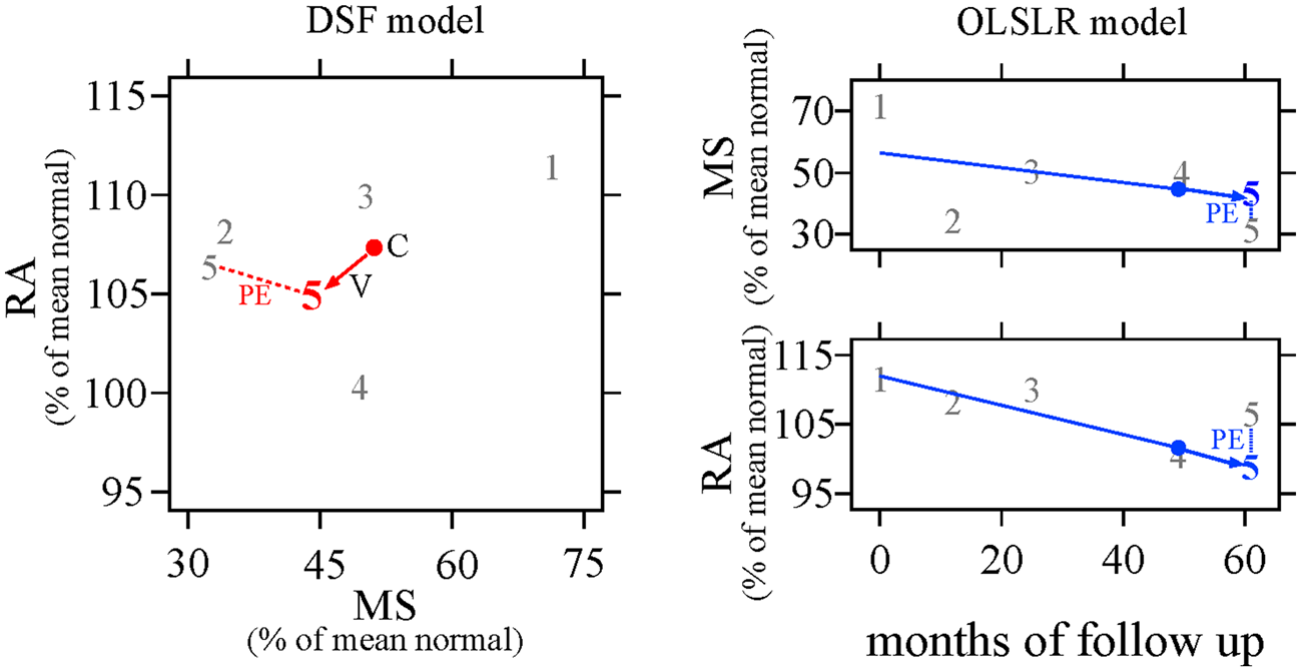
Illustration of the prediction of structure–function pairs by the DSF model (left panel) and by OLSLR (right panel). In the left panel, the DSF model is depicted in two–dimensional space with MS on the x–axis and RA on the y–axis (both expressed as % of mean normal). Numbers 1–5 (in gray text) represent the observed RA and MS measurements at the 1st to 5th visit. To predict the values of RA and MS at the 5th visit with the DSF, the first 4 observed RA–MS pairs are used to estimate the centroid (C, solid red circle) and velocity vector (V, red arrow), which are in turn used to predict the paired measurement at the 5th visit (number 5 in red text). In the right panel, the first four series of observed RA and MS data are plotted separately over time. For OLSLR prediction of RA and MS values at the 5th visit (number 5 in blue text), the expected value is estimated from the best fit line for each series, as shown with the blue arrow. For both models, the error in prediction is estimated by comparing the predicted measurements (colored “5 s”) to the observed measurement (gray “5 s”).

In the current study, the DSF and OLSLR models were independently applied to predict future RA–MS paired measurements from the OHTS–CSLO dataset. The two models were also used to predict future RNFLT–MD paired measurements from the resampled DIGS/ADAGES dataset. The section below provides a description of how each model was used to predict future structure–function measurements. A detailed description of the DSF model is available in Hu et al.^26^. In Fig. 5, we briefly describe how the DSF model and OLSLR were applied to predict RA–MS paired measurements at the 5th visit.

#### Predictions by DSF model

The DSF model employs two vectors: a centroid and a velocity vector, to predict future structure–function paired measurements from preceding data. Whereas the centroid is an estimate of the current stage of the disease (the central location of the series of observed structure–function paired measurements), the velocity vector is a measure of the direction and speed at which the structure–function pairs are changing over time. Consider RA–MS paired values (*X*1, *X*2, *X*3 and *X*4) measured over four visits with time (*t*) intervals points: *t*1, *t*2, *t*3 and *t*4. To predict the RA–MS pair at the 5th visit (at time *t*5) by the DSF model, first, the arithmetic mean for the first four observed data pairs is calculated as the centroid(*C*); $C=\frac{\left(X1 + X2 + X3 + X4\right)}{4}$. The model then determines the velocity vector(*V*), which is computed as an average of all rates of change from visit to visit. Thus, $V=\frac{\left(X2-X1\right)}{\left( t2-t1\right)}+\frac{\left(X3- X2\right)}{\left( t3-t2\right)}+ \frac{\left(X4- X3\right)}{\left( t4-t3\right)}$. The expected paired values at the 5th visit *(P*) are derived by adding the paired values at the current state of the disease (centroid) and the average change in paired measurements. This is mathematically represented as $P=C+\left(\frac{V}{ t5-t4}\right)$. As, shown in the left panel of Fig. 5, the predicted measurements are then compared to the observed values for the RA–MS pair at the 5th visit to estimate the error in prediction.

#### Predictions by OLSLR model

OLSLR predictions were derived by fitting the model separately to the available series of structural and functional measurements. For example, to predict RA and MS measurements at the 5th visit, OLSLR was fitted separately to the first four RA measurements and to the first four MS measurements. The expected measurements at the 5th visit were estimated from the best fit lines for the RA and MS series, as shown in the right panel of Fig. 5.

### Statistical analysis

The prediction accuracy for each model was assessed by determining the magnitude of the resulting PE in percent of mean normal. The magnitude of the PE was computed as the square root of the sum of the squared differences between the predicted value and the observed value for each component of the structure–function pair. Predictions were from the 4th to 7th visit for global and sectoral RA–MS pairs, and for RNFLT–MD pairs. For each category of prediction, the Wilcoxon signed rank test was used to determine whether the difference in median PE between the DSF model and OLSLR was statistically significant. Significance level was set at 0.05. All analyses were carried out in R^50^ and SPSS (version 26.0; IBM, Armonk, NY, USA).

## Data Availability

The datasets analyzed in the current study are not publicly available due to data sharing agreement issued by the primary sources of the two datasets. Information for submitting requests to access datasets from these studies is available from the corresponding author.
